# Classifying driver mutations of papillary thyroid carcinoma on whole slide image: an automated workflow applying deep convolutional neural network

**DOI:** 10.3389/fendo.2024.1395979

**Published:** 2024-11-06

**Authors:** Peiling Tsou, Chang-Jiun Wu

**Affiliations:** Department of Genomic Medicine, University of Texas, MD Anderson Cancer Center, Houston, TX, United States

**Keywords:** papillary thyroid carcinoma, driver mutations, whole slide images, convolutional neural network, digital pathology, deep learning

## Abstract

**Background:**

Informative biomarkers play a vital role in guiding clinical decisions regarding management of cancers. We have previously demonstrated the potential of a deep convolutional neural network (CNN) for predicting cancer driver gene mutations from expert-curated histopathologic images in papillary thyroid carcinomas (PTCs). Recognizing the importance of whole slide image (WSI) analysis for clinical application, we aimed to develop an automated image preprocessing workflow that uses WSI inputs to categorize PTCs based on driver mutations.

**Methods:**

Histopathology slides from The Cancer Genome Atlas (TCGA) repository were utilized for diagnostic purposes. These slides underwent an automated tile extraction and preprocessing pipeline to ensure analysis-ready quality. Next, the extracted image tiles were utilized to train a deep learning CNN model, specifically Google’s Inception v3, for the classification of PTCs. The model was trained to distinguish between different groups based on *BRAF^V600E^* or *RAS* mutations.

**Results:**

The newly developed pipeline performed equally well as the expert-curated image classifier. The best model achieved Area Under the Curve (AUC) values of 0.86 (ranging from 0.847 to 0.872) for validation and 0.865 (ranging from 0.854 to 0.876) for the final testing subsets. Notably, it accurately predicted 90% of tumors in the validation set and 84.2% in the final testing set. Furthermore, the performance of our new classifier showed a strong correlation with the expert-curated classifier (Spearman rho = 0.726, p = 5.28 e-08), and correlated with the molecular expression-based classifier, BRS (BRAF-RAS scores) (Spearman rho = 0.418, p = 1.92e-13).

**Conclusions:**

Utilizing WSIs, we implemented an automated workflow with deep CNN model that accurately classifies driver mutations in PTCs.

## 1 Introduction

Thyroid cancer is the most prevalent type of endocrine malignancy (1). Papillary thyroid carcinoma (PTC), which accounts for nearly 80% of all thyroid cancers, is a mitogen-activated protein kinase (MAPK) -driven malignancy characterized by two mutually exclusive driver mutations: *BRAF^V600E^* and mutated *RAS*, each triggering different downstream signaling events (2). The *BRAF^V600E^* mutation is present in almost half of all PTC cases. *RAS* point mutations, affecting specific loci (codons 12, 13, and 61) in the N-RAS, H-RAS, or K-RAS genes, occur in 10% to 15% of PTC patients (3). Notably, the *BRAF^V600E^* mutation not only correlates with increased tumor aggressiveness (4) but also hampers the tumor’s ability to uptake radioactive iodine (RAI) (5), resulting in unfavorable prognoses (6, 7). Tumors with the *BRAF^V600E^* mutation do not respond to the ERK-mediated negative feedback on RAF, causing elevated MAPK signaling (8). Conversely, tumors driven by *RAS* activate RAF dimers that are sensitive to ERK (extracellular signal-regulated kinases) negative feedback, which in turn decreases MAPK signaling. These differences in signaling mechanisms lead to significant phenotypic divergence, which could be crucial for therapeutic or prognostic strategies.

Recent advancements in artificial intelligence have facilitated the use of various imaging modalities for early detection of malignancies (9) as well as the implementation of digital pathology in precision oncology (10–15). Current mutation detection techniques, such as immunohistochemistry, real-time polymerase chain reaction (PCR) and automated platforms all require a substantial amount of tumor tissue for analysis (16). Recently, image-based analysis has demonstrated great potential in predicting mutations (13, 17, 18), which is especially useful when the tumor sample is insufficient for direct testing. Our work (19), along with research from various groups (16, 20–23), has shown that deep CNN models can predict actionable gene mutations from histopathologic images. While it is improbable that deep learning-based mutational predictions will soon replace direct molecular testing of tissue samples, these computational techniques can offer vital insights to pathologists and oncologists, assisting in clinical management decisions and helping prioritize patients for comprehensive sequencing.

However, the manual examination of slide images remains a tedious process prone to variability and bias among different raters. Tasks like tissue segmentation and preprocessing of whole slide image (WSI) are crucial in automated digital pathology workflows. Recently, the U.S. approval of the first WSI scanner for primary diagnosis has marked a significant step forward in integrating digital pathology into clinical practices (24, 25). WSI technology has improved archiving efficiency, facilitated remote diagnosis, and accelerated clinical judgments and research processes (12, 13, 26).

In this study, we sought to expand upon our previous work (19) by developing an automated pipeline. This new system begins with a tile-selection mechanism applied to raw WSIs, continues with image preprocessing steps, and concludes with a classifier for driver mutations.

## 2 Materials and methods

### 2.1 Sample selection

We obtained WSIs, genomic data, and corresponding demographic and clinical information for matched patient samples from the TCGA website (https://gdc.cancer.gov/). Although each patient might have several slides available, we chose to analyze only one diagnostic slide for each patient. The tumor tissue was hematoxylin and eosin (H&E)-stained, fixed in formalin, and embedded in paraffin (FFPE). An Aperio SVS file stores an image at multiple resolutions, including the highest resolution at which the section image was captured. These SVS files were downloaded in their native format.

**Figure 1:**
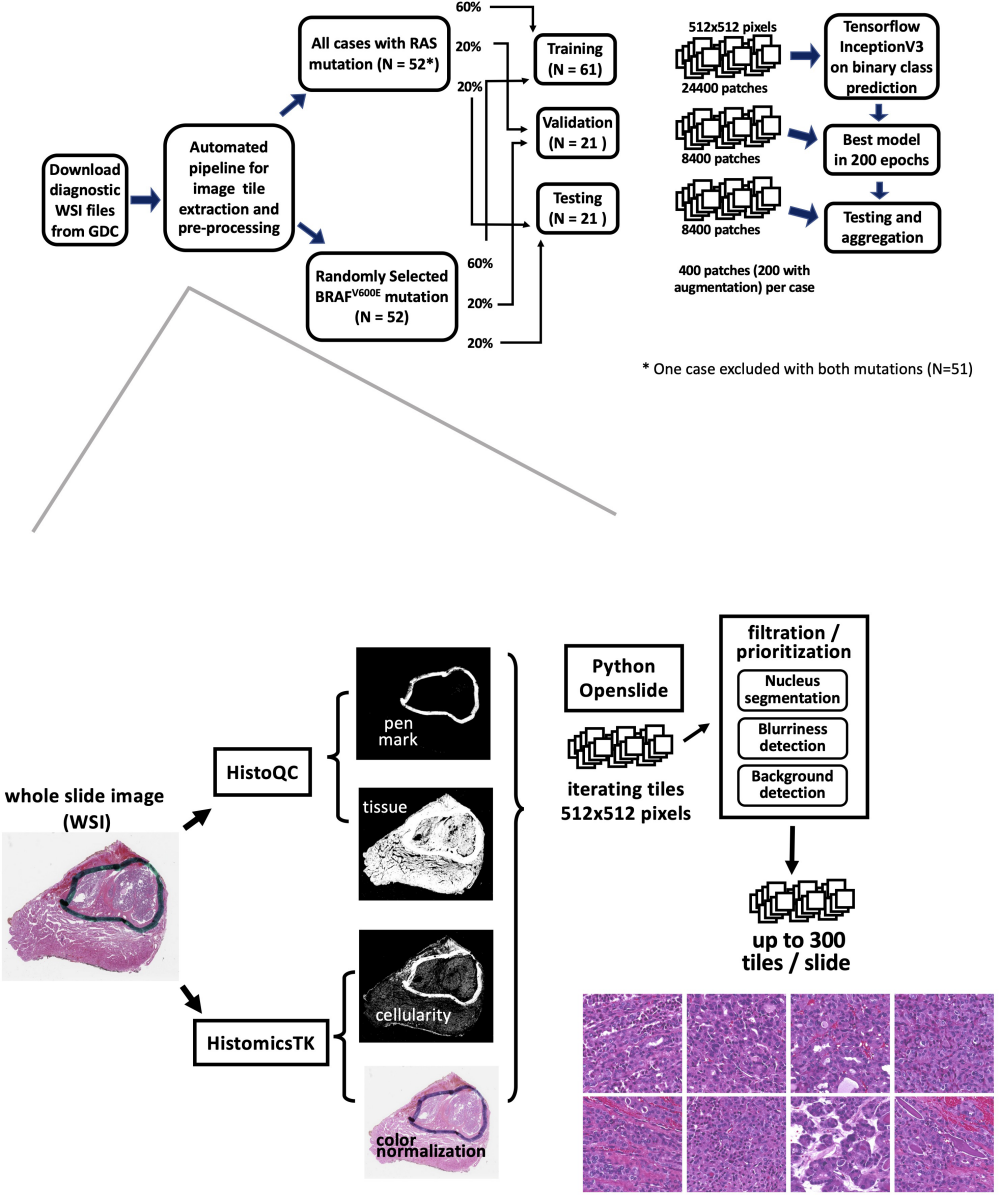
The workflow of an automated mutation classifier using WSI. The upper panel illustrates the sample selection, allocation, and analysis strategy. Hematoxylin and eosin (H&E)- stained images of formalin-fixed, paraffin-embedded (FFPE) slides were obtained from the GDC repository. Samples exhibiting *RAS* or *BRAF^V600E^* mutations were distributed into training, validation, and testing sets at ratios of 60%, 20%, and 20%, respectively. The flow for extracting image tiles and the preprocessing steps is depicted in the bottom panel.

A total of 235 samples exhibited *BRAF^V600E^* mutations, while 52 samples showed RAS hotspot mutations. However, one sample (TCGA-EM-A4FV) was identified as harboring both mutations and was consequently excluded from the analysis. The histological classification distribution for all *BRAF^V600E^* and *RAS* mutation samples can be found in **Supplementary Table S1**. Notably, all 51 *RAS* mutation samples were included in the study. Conversely, 52 samples were randomly chosen from the 234 cases with *BRAF^V600E^* mutations, ensuring a general match in the distribution of histological types (**Figure 1**). The characteristics of the patients are detailed in **Supplementary Table S1**. Patients were randomly assigned to distinct groups for training (60%), validation (20%), and final testing (20%). These sets were used for training the model and evaluating its performance.

Tumors carrying different driver mutations exhibit distinct distributions of pathological classification. **Supplementary Table S1** indicates that classical PTCs are the predominant type in cases with the *BRAF^V600^* mutation, comprising 76.5% of the cases. Additionally, 5.1% are classified as the follicular variant, and 11.5% as the tall cell variant. In tumors with *RAS* mutations, pathological assessments were conducted on 49 out of 51 samples, identifying 29 as follicular variant and 20 as classical PTCs.

### 2.2 Tile extraction and image preprocessing

We implemented an automated image tile extraction approach to select high-quality and high-cellularity patches. Briefly, we first used the HistoQC package (27) to identify tissue regions on the WSI and mask out low-quality regions such as blood, bubbles, blurred regions, or pen marks. Next, color normalization was applied to the tissue region on the whole slide. Subsequently, we iterated over the WSI for candidate 512x512 pixel patches at 20X magnification and implemented a nuclear segmentation python package HistomicsTK (28) to characterize cellular nuclei and calculate the numbers, staining density, and dimension ratios of nuclei in the patch. Up to 300 image patches were extracted from each WSI. The details of our automatic workflow are described in **Supplementary Table S2B**. Two hundred image patches were randomly chosen from preprocessed patches per sample for model training. We subsequently implemented an image augmentation technique on the training data. By applying vertical and horizontal flips, along with 90-degree rotations, we generated 8 possible variations for each patch, from which two were randomly selected for the training data. No augmentation was applied for validation and final testing tile images.

### 2.3 Network architecture and training, validation, and final testing procedures

Our imaging classification model was derived from a modified Inception v3 architecture (29), with Adam optimizer (learning rate of 0.001) and final softmax activation layer implemented with Tensorflow in Python. The classifier processes a 512x512x3 image tile as input and outputs the probability that the tile originates from an RAS-mutated tumor. We adapted the model to facilitate a two-label output classification. The architecture of the model is described in **Supplementary Table S2A**.

Patients were randomly allocated to non-overlapping training (60%), validation (20%), and final testing (20%) subsets (**Figure 1**). There were 24,400 patches for training, 4,200 for validation, and 4,200 patches for final testing. In total, our model was trained through 200 epochs, equivalent to 610,000 iterations with a batch size of 8. At the end of each epoch, log-loss and accuracy on the validation data were calculated. The final model was selected based on the lowest cross-entropy loss observed in the validation data.

### 2.4 Performance evaluation

#### 2.4.1 Evaluation metrics for algorithm performance

We assessed the performance of our CNN model using receiver operating characteristic (ROC) curves, where a higher area under the ROC curve (AUC) indicates superior prediction capability. The significance (p-value) of the predictions was determined through Fisher’s exact test. Additionally, we utilized confusion matrices to summarize prediction accuracy.

#### 2.4.2 percRAS score

The final softmax layer of our CNN model computed the probability of *BRAF^V600E^* or *RAS* mutations for each input image tile. With the automatic extraction pipeline, each tumor generates multiple tiles, which are inputted into the classification network. While evaluating performance tile by tile is straightforward, assessing performance at the tumor level can be approached in various ways. Our approach involved utilizing all tiles associated with a patient sample and determining the fractions of the two mutation types as the class probabilities for that sample. An image tile was categorized as RAS if the probability predicted by the model for RAS mutant was 50% or higher. At the tumor sample level, predictions of mutation classes were based on a probability cutoff level of 0.75. For each tumor, a percRAS score was calculated as the percentage of tiles predicted as RAS mutation class. We classified a tumor as *RAS* mutated if the percRAS score exceeded 0.75, and as *BRAF^V600E^* mutated if the percRAS score was below 0.25. If the percRAS score fell between 0.25 and 0.75, the mutation class was not predicted.

#### 2.4.3 BRAF-RAS score

The BRS values were derived from the landmark research of TCGA (2), which involved comparing 391 samples with both exome and RNA sequencing data to establish a 71-gene signature. This signature was used to calculate correlations, resulting in a continuous measure ranging from -1 to +1, where BRAF-like (BVL) PTCs exhibit negative values and RAS-like (RL) PTCs display positive values.

## 3 Results

### 3.1 An automated pipeline for image tile extraction and preprocessing

Using expert-guided patch selection, we previously obtained decent results (19), comparable to recent studies by other researchers who used the TCGA resource to investigate different types of cancer (20, 30). To compare the performance of the newly developed automatic image preprocessing pipeline, we utilized the same samples, CNN framework, and analysis strategies as shown in the upper panel of **Figure 1**. Furthermore, to automate the selection and preprocessing of high-quality, high-cellularity patches from WSIs, we developed a workflow, as depicted in the lower panel of **Figure 1**.

### 3.2 Deep learning models derived by automated workflow effectively differentiated BRAF^V600E^ and RAS mutation using WSIs in PTCs

**Figure 2:**
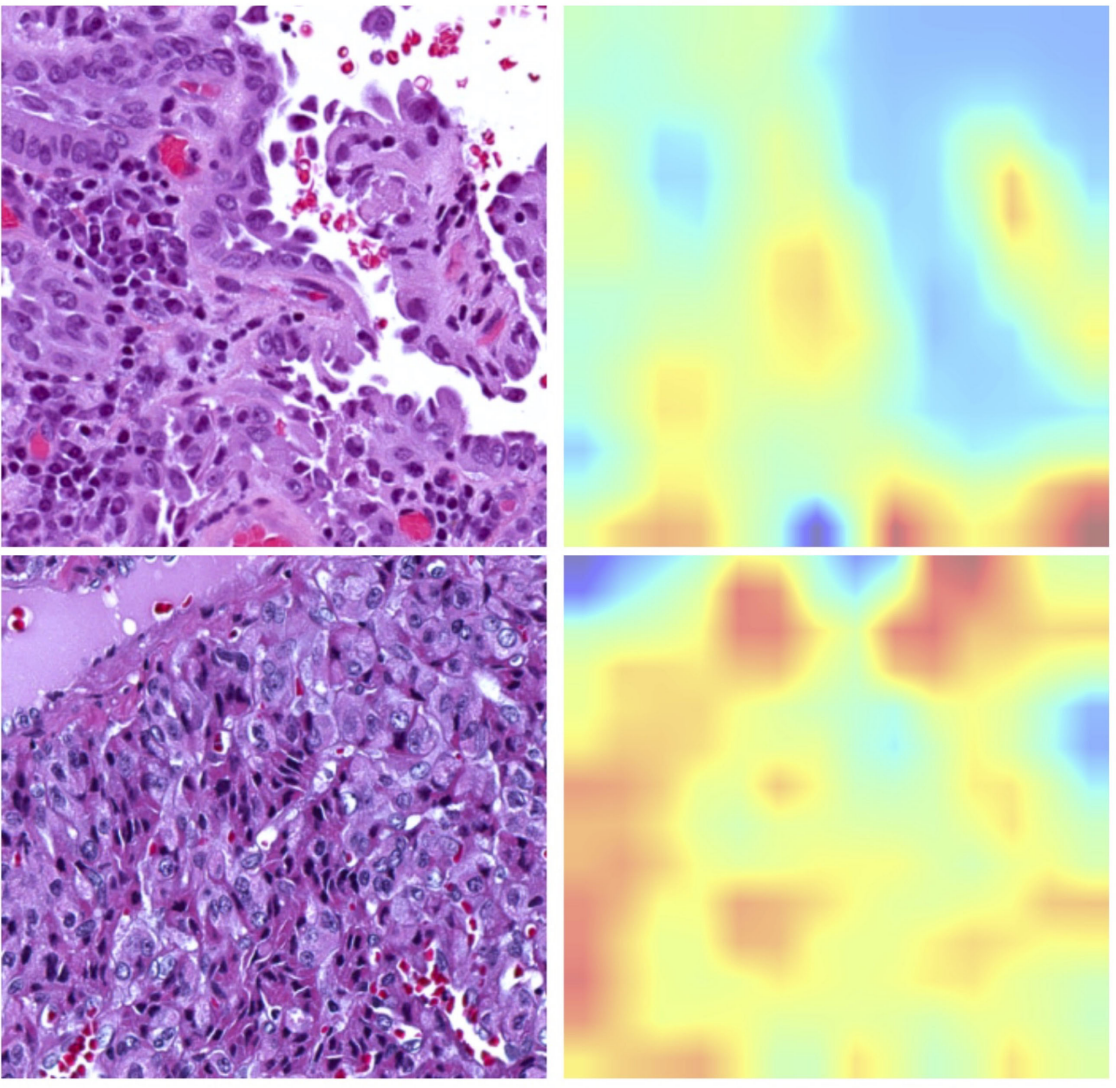
Representative histopathological images alongside corresponding activation maps for *RAS* or *BRAF^V600E^* mutations. The upper panel displays images from *BRAF^V600E^* mutated samples, showcasing the Classical Histological Type. The bottom panel features images from *RAS* mutated samples, illustrating the Follicular Variant Type.

**Figure 3:**
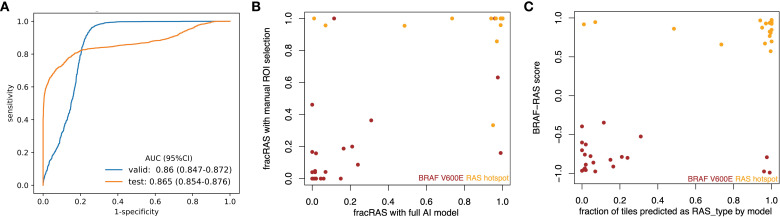
CNN models effectively differentiated *BRAF^V600E^* and *RAS* mutations in PTCs based on histopathological images. **(A)** ROC curves for the best model showed AUC values of 0.86 for the validation subset and 0.865 for the final testing subset at 20X magnification. **(B)** The results from the new classifier showed a strong correlation with those from an expert-curated classifier using manual ROI selection (Spearman rho = 0.726, p < 0.001). **(C)** The outcomes from the new classifier correlated well with those from BRS, a molecular expression-based classifier (Spearman rho = 0.556, p < 0.001).

The newly developed pipeline effectively differentiated between *BRAF^V600E^* and *RAS* mutations using WSIs in PTCs. **Figure 2** displays representative histopathological images along with activation maps of *BRAF^V600E^* and *RAS* mutations. As described in **Figure 3A**, the best model achieved AUC values of 0.86 for validation and 0.865 for final testing subsets. Overall, the model accurately predicted 90% of validation cases and 84.2% of the final testing tumor samples.

In line with our previous findings and those of others (2, 19), the prediction demonstrated better performance in detecting *RAS* mutations compared to *BRAF^V600E^* mutations. In the final testing subset, the prediction accuracy was 70% for the *BRAF^V600E^* group and 100% for the *RAS* group. **Supplementary Table S3** provides detailed confusion matrices highlighting the disparities among various classifications.

### 3.3 The performance from the automatic image extraction and preprocessing pipeline was comparable to that of an expert curated ROI approach

With expert-guided patch selection, we previously achieved solid results with an AUC of 0.878 – 0.951, comparable to recent work on other cancer types using TCGA resource. Using the same samples and deep CNN framework, the performance (**Figure 3A**) by automated preprocessing pipeline slightly declined (AUC 0.860 – 0.865). We further checked to see whether the classification results from these two classifiers were consistent. Compared to the expert-curated Region of Interest (ROI) classifier, the automated tile extraction and preprocessing classifier achieved consistent results (Spearman rho = 0.726, p = 5.28 e-08), as shown in **Figure 3B**.

### 3.4 Analyses from images correlate well with BRS

Previously, we showed that the expert-curated classifier had a strong correlation with BRS, a pattern based on mRNA expression (19). We have now assessed the new classifier to determine its correlation with mRNA expression data. As shown in **Figure 3C**, there was a significant correlation (Spearman’s rho = 0.418, p = 1.92e-13) between mRNA-based BRS and the information from histopathology images. Specifically, of the 180 tumors identified as BRAF-like (BRS < 0), 145 were categorized into the *BRAF^V600E^* group. Additionally, 45 out of 48 tumors identified as RAS-like (BRS > 0) were classified with RAS mutations. **Supplementary Table S3** includes confusion matrices that highlight the differences among the various classifications.

## 4 Discussion

CNN techniques remain the state of the art in applying deep learning to histopathology (31). Several CNN models have been deployed in classifying tissue images in cancer research, including AlexNet, ResNet-101, VGG16/19, Inception v3 and other networks (31, 32). Different architectures excelled in different specific tasks (32). Inception v3 was used in a study to classify mutation types in NSCLC with high accuracy (20). We followed our previous work (19) and used the Inception V3 architecture in the current driver mutation prediction study.

The current study described an automated workflow that applied deep learning approaches to identify driver mutations from WSIs. In our previous work (19), we effectively showcased the potential of correlating genomic information with histopathologic images. However, our approach did not incorporate automated WSI segmentation. Instead, it involved labor-intensive selection of ROIs within each slide, requiring expert guidance. In this study, we introduced automatic image tile extraction and preprocessing to enhance efficiency and include a larger portion of each slide in training and prediction processes.

It has been shown that an image-based classifier trained with mutation-stratified slides performs well in distinguishing tumors with different oncogene expression patterns (10, 33). Similarly, our classifier, which was trained on samples stratified by mutations, demonstrated a strong correlation between image-based classification and mRNA expression patterns (BRS). It is likely that these specific mutations influence downstream signaling pathways. Therefore, both mRNA expression profiles and the structural features observed in histopathology comprehensively reflect these molecular processes.

In our previous work (19), we noticed that our CNN models were more effective at identifying RAS mutations than BRAF mutations, and the same finding was also observed in the newly developed pipeline. This aligns with the observations of a recent landmark TCGA study on PTCs (2). The study highlighted fundamental differences in the genomic, epigenomic, and proteomic profiles between RL-PTCs and BVL-PTCs. Notably, it also recommended that *BRAF^V600E^* PTC should not be treated as a uniform group in clinical research.

The advancement of AI techniques has significantly accelerated the resolution of complex problems (34). With the recent FDA approval of the first WSI system for primary diagnosis in pathology (24, 25), digital pathology using WSI analysis appears set for integration into standard clinical practice. While the performance of our automated workflow, which did not require expert-annotated ROIs, was slightly lower compared to our previous deep CNN classifier, it still achieved favorable AUCs and overall accuracy. The classification results were in good agreement with both the expert-curated and molecular classifiers (BRS). Additionally, this approach offers significant benefits, such as eliminating the need for labor-intensive manual annotations, reducing the time and cost associated with data preprocessing, and decreasing inter-rater bias and variability.

Due to the limited number of cases studied, there is potential for improvement in our current work. Initially, as a proof of concept, we focused only on the two predominant mutations found in PTCs: *BRAF^V600E^* and *RAS*, which together are estimated to account for over two-thirds of PTC cases. However, without a multi-classifier, the clinical applicability of our findings remains somewhat restricted. Future efforts should explore genomic alterations beyond these two mutations. Additionally, the concept of intra-tumoral heterogeneity should be considered to provide a more comprehensive understanding. Last but not least, real-world pathology practices vary significantly in terms of case mix, patient demographics, and diagnostic protocols. An automated system that performs well in one clinical setting may not generalize to others without additional validation and fine-tuning. Thus, while automated pipelines for digital pathology in WSI offer promising advancements, addressing biases and ensuring the generalizability of the system in real-world clinical settings requires careful consideration. Despite these limitations, we believe that our automated workflow using deep CNNs represents a step forward, potentially aiding molecular pathology and offering valuable clinical insights. Further efforts in improving data diversity by incorporating diverse and representative data, cross-institutional validation, continuous learning and adaptation will be essential for realizing the full potential of these technologies in improving patient care.

## Data Availability

Publicly available datasets were analyzed in this study. This data can be found here: https://portal.gdc.cancer.gov/projects/TCGA-THCA.

## Supplementary material

Supplementary Table 1 Demographic characteristics and histological types of the PTC.

Supplementary Table 2 Detailed description of the Inception v3 CNN model and the automatic workflow.

Supplementary Table 3 Confusion matrices detailing the prediction outcomes from the automated workflow.
